# Reflecting on gamified learning in medical education: a systematic literature review grounded in the Structure of Observed Learning Outcomes (SOLO) taxonomy 2012—2022

**DOI:** 10.1186/s12909-023-04955-1

**Published:** 2024-01-03

**Authors:** Wenhao David Huang, Viktoria Loid, Jung Sun Sung

**Affiliations:** 1 Biomedical and Translational Science, Carle-Illinois College of Medicine; Education Policy, Organization, and Leadership, College of Education, University of Illinois Urbana-Champaign, Champaign, IL USA; 2 Education Policy, Organization, and Leadership College of Education, University of Illinois Urbana-Champaign, Champaign, IL USA

**Keywords:** Medical education, Gamification, Gamified learning, SOLO taxonomy, Learning outcomes

## Abstract

**Background:**

The acquisition of in-depth medical knowledge, skills, and competencies is of utmost importance when training future medical professionals. This systematic literature review delves into the empirical connection between gamified learning in medical education and the Structure of Observed Learning Outcomes (SOLO) taxonomy.

**Methods:**

Following PRISMA guidelines, a systematic literature review was conducted in seven databases to identify empirical studies related to gamification and medical education. The literature search was limited to peer-reviewed articles published between January 2012 and December 2022. Articles focusing on games or learning technologies in a broader sense as well as research focusing on nursing or pharmacy education were excluded.

**Results:**

Upon reviewing 23 qualified empirical studies that applied gamified learning strategies in medical education, 18 (78%) studies are associated with the second lowest level (uni-structural) of the SOLO taxonomy. The mid-level (multi-structural) learning outcomes are associated with three (13%) of the reviewed studies. There are five (22%) studies focusing on the second highest (relational) level of the SOLO. Only one study (4%) is associated with the highest SOLO level (extended-abstract). Finally, three (13%) studies were identified with multiple levels of the SOLO. In addition to the SOLO levels, the review found six (26%) studies emphasizing motivational and engagement support of gamified learning strategies in facilitating intended learning outcome attainment. A total of three (13%) studies, across three SOLO levels, suggested that gamified learning strategies can improve students’ soft skills in medical education programs.

**Conclusion:**

These findings collectively emphasize the need for future research and development to design gamified learning experiences capable of fostering higher SOLO taxonomy attainment in medical education. Moreover, there is potential to extend the SOLO framework to encompass motivational and affective learning outcomes, providing a comprehensive understanding of the impact of gamified learning on medical students.

## Background

### Rationale

The overarching goal of medical education is to equip students with the ability to perform in their future profession [1]. The acquisition of in-depth medical knowledge, skills, and competencies is of utmost importance when training future medical professionals. Future health professionals should be able to correctly diagnose patients, make informed decisions about suggested treatments, and communicate these medical procedures to patients [2–4]. In addition, competencies in clinical reasoning [5] and awareness of the need for the global health workforce [6] are included in recent systematic literature review studies. Although defining medical education is beyond the scope of this study, it is important to recognize the collective nature of medical education grounded in many educational assumptions, inlcuding experiential learning, reflective learning, competency-based learning, problem-based learning, structured assessments, and the use of technologies to enhance learning outcomes [7].

Systematic reviews on the efficacy of health professional and medical education have offered diverse perspectives on the potential effects of various educational and learning interventions. We identified four needs to justify the need for the present study. First, there is a need for theoretically grounded research in the health profession and medical education. Eskander et al. reviewed 18 studies on the impact of wellness interventions for resident physicians and suggested the need to reference education theories for future program development in similar areas for optimal educational outcomes [8]. In the context of graduate medical education. In a review of 132 studies, Song and colleagues proposed research design changes to employ a theoretical framework to guide ophthalmology education research [9]. Second, there is a need for systematic and guided approaches to better understand the quality of health professional and medical education. De Leeuw and colleagues reviewed 418 publications that evaluated postgraduate medical e-learning [10]. Their review implies the need for a consistent, validated, and guided evaluation approach to help medical educators understand the complexity of e-learning in graduate medical education. Vasquez and colleagues reviewed 24 studies on the effects of skill-based programs on reducing physician burnout [11]. Additional future research using randomized controlled trials was suggested in their review. Under the influence of the COVID-19 pandemic, Wilcha reviewed 34 studies that focused on the effectiveness of virtual medical teaching [12]. This review identified several factors that impact the quality of virtual medical teaching, including reduced learner engagement and the absence of necessary learning assessments due to technological limitations.

Third, interest in educational and instructional strategies for health professional and medical education is heterogeneous because they serve a wide range of potential educational outcomes. Aldriwesh et al. proposed a global interprofessional education approach to better meet the need of a global health workforce based on a review of 16 peer-reviewed studies [6]. Grounded in 21 studies, the effect of teaching methods on evidence-based practice is inconclusive [13] because of the lack of homogenous approaches in assessing intended learning outcomes (knowledge, skills, attitudes, and behaviors). Fourth, there is a need to better understand the gamification of learning in health and medical education. A short online gamified learning activity could be helpful in supporting learners’ confidence in health professional training when face-to-face interactions are infeasible [14]. In a randomized experimental study to compare the efficacies of various learning activities (i.e., e-module, e-modules + cases, and e-modules + cases represented by high-fidelity simulation games), gamified learning reportedly engaged learners cognitively more than the case group [15]. Upon identifying 49 studies to review, Vermeir et al. confirmed the effects of gamification on learner motivation and engagement during cognitive training [16]. Scott and colleagues indicate the importance of allowing students to practice complex tasks in realistic situations to adequately prepare them for the challenges of the medical profession [4]. Further, Middeke et al. suggest that gamified educational methods allow students to improve their clinical reasoning skills [3]. The positive clinical effects of gamification have also been reported in multiple recent review studies in the context of rehabilitation or physical training [17–19]. The systematic literature review by van Gaalen et al. on the gamification of health professional education captures the rationale for the present study to be the best [20]. Based on 44 studies, their review suggests an emerging need to delineate the conceptual, pedagogical, and empirical alignment between the features of gamification and their effects on intended learning outcomes. To address the above four needs with a parsimonious and homogeneous literature review approach grounded in education theories, this present study reviews the recent literature on the gamification of health professions and medical education in achieving specific cognitive learning outcomes.

### Selection of learning outcome framework

The overarching goal of medical education is to equip students with the ability to perform their future professions [1, 21]. This suggests that the acquisition of in-depth medical knowledge, skills, and competencies is of utmost importance when training future health and medical professionals. Future health professionals should be able to correctly diagnose patients, make informed decisions about suggested treatments, and communicate these medical procedures to patients [2–4]. Although medical education involves a range of learning outcomes [22] ranging from the pure acquisition and retention of knowledge [23] to the ability to correctly perform and communicate in high-pressure situations [24], effective clinical performance must first be supported by the acquisition of factual knowledge, knowledge application, and clinical reasoning skills [1, 21, 23, 25]. Therefore, cognitive reasoning is the primary learning outcome, which leads to the adoption of The Structure of Observed Learning Outcomes (SOLO) taxonomy [26] for this review.

The SOLO taxonomy, derived from observed assessments, is primarily focuses on the cognitive learning outcomes associated with various subject matters [26]. More importantly, by comparison with Bloom’s Taxonomy [27], SOLO taxonomy is situated in learners’ developmental “modes of learning” grounded in Piaget’s scholarship on cognitive development (i.e., sensorimotor, iconic, concrete symbolic, formal, and post-formal) [28] and the cyclic process for learners to attain certain mode of cognitive development [28]. In other words, Bloom’s Taxonomy provides clear and concrete learning outcomes as goals for developing effective learning objectives, while SOLO taxonomy helps educators consider learners’ internal cognitive developmental processes when designing intended educational interventions. The SOLO taxonomy includes five cyclic levels of progressively arranged learning outcomes within each mode of learning [28–30]:

- Pre-structural is pre-learning, in which learners use irrelevant and unorganized data with no effects in solving problems. This level concerns learners’ ignorance and lack of usable knowledge.
- Uni-structural is when learners use only one piece of relevant data to solve problems. These results may be inconsistent. This level can lead to learners' ability to recall, memorize, or define knowledge.
- Multi-structural is when learners use multiple data pieces to solve problems without integrative relationships among them. The problem-solving results remain inconsistent. This level refers to learners’ ability to gather, combine, and list concrete knowledge facts.
- Relational refers to when learners use all available data points, with an understanding of the relationships among them, to form an applicable problem-solving system with consistent results. This level deals with learners’ abilities to analyze, compare, relate, and explain.
- Extended Abstract is when learners make connections with data, information, and relationships beyond the initial problem-solving context to enable the transfer of learning and performance from one situation to another. This level develops learners’ ability to reflect, hypothesize, or theorize.

The second and third levels are focused on the quantity of knowledge that learners can internalize and retrieve. The final two levels of the SOLO taxonomy relate to learners’ abilities to work with abstraction [31]. All levels articulate the cyclic nature of cognitive learning outcomes in order to achieve certain modes of learning. Learners can be in multiple SOLO levels simultaneously [28, 29] while attending to both surface and deep learning approaches [32–34].

The SOLO taxonomy has been applied to align medical and dental education learning outcomes with respective learning or instructional strategies. Pandey and Zimitat applied SOLO taxonomy to understand the learning of human anatomy among first-year medical students (*n* = 97) and found that learners’ SOLO ratings correlated positively with their deep learning scores based on a version of the Study Process Questionnaire (*r* = 0.24, *p* < 0.01) and positively with learners’ final grades (*r* = 0.61, *p* < 0.01) [33]. To optimize the cognitive alignment between study materials and learners’ abilities to internalize knowledge on anatomy, D’Antoni et al. analyzed the primary learning outcomes of studying human anatomy against the SOLO taxonomy from the perspective of cognitive information processing [35]. Given the information-heavy nature of the subject matter, the authors suggested that learners avoid rote learning strategies (i.e., highlighting information from the study materials and rereading the study materials). Both learning strategies are categorized as early levels of SOLO taxonomy with limited effects on the development of long-term memory. Instead, learners should self-test their retrieval of intended knowledge (i.e., practice testing) [35]p159 and distribute their learning and testing over time to facilitate information internalization (i.e., distributed practice) [35]p161. Both strategies are focused on efficient cognitive learning with the purpose of passing pertinent cognitive tests on human anatomy. Reid and colleagues designed a collection of assessments based on the SOLO taxonomy for 3rd-year medical students, which includes multiple-choice questions, short-response questions, structured case study narratives, and peer assessment [34]. Upon completing the assessments, the learners completed the Approaches to Study Skills Inventory for Students for their surface or deep learning strategies. The results demonstrated positive correlations between higher levels of SOLO-based assessment and higher deep learning scores. Svensäter and Rohlin also applied the SOLO taxonomy to develop a formative and summative assessment model for dental students [30]. They reported the feasibility of SOLO taxonomy levels for guiding assessment development. Regardless of subject matter, learning outcomes should guide the selection and deployment of corresponding teaching and instructional strategies to ensure the attainment of intended learning objectives [36]. Learning outcomes should be the primary reference point for all educational and instructional events. Not just assessments.

### Gamified learning for learning outcome attainment

Recent reviews of the achievement of learning outcomes in medical education have found a lack of learning transfer among medical students [37, 38]. Scott and colleagues indicated the importance of allowing students to practice complex tasks in realistic situations to adequately prepare them for the challenges of the medical profession [4]. Across subject matter areas, gamified learning, as an instructional strategy that incorporates gameplaying elements into intended learning processes [39], has been applied to facilitate knowledge transfer and scaffold the attainment of complex cognitive learning outcomes [40, 41]. Beyond cognitive learning outcomes, Huang et al. validated empirical relationships between features of gamified learning and learners’ motivation to learn blood types among undergraduate students [42]. In health sciences education, McKenzie compared the impact of a face-to-face teaching event with a short online game-informed learning (GIL) activity and revealed the positive effects of GIL on learners’ perceived confidence, interest level, and clarity of intended learning [14]. Middeke et al. suggested that gamified educational methods allow students to improve their clinical reasoning skill set [3]. Nevertheless, through a systematic review of the effects of gamification in health professions education, Gentry et al. called for further reviews grounded in theoretical perspectives [43].

### Objectives

The study, through reviewing the literature on gamified learning and its targeted learning outcomes in medical education grounded in the SOLO taxonomy from 2012 to 2022, empirically associates gamified learning and the SOLO taxonomy in the context of medical education. The scope of medical education for this study includes all undegradaute, gradaute, and continuous medical education programs that train healthcare professionals in a diverse clinical settings [7]. The findings of this study can inform the adoption of gamified learning strategies to help medical students attain cognitive learning outcomes grounded in the SOLO taxonomy.

## Methods

**Fig. 1:**
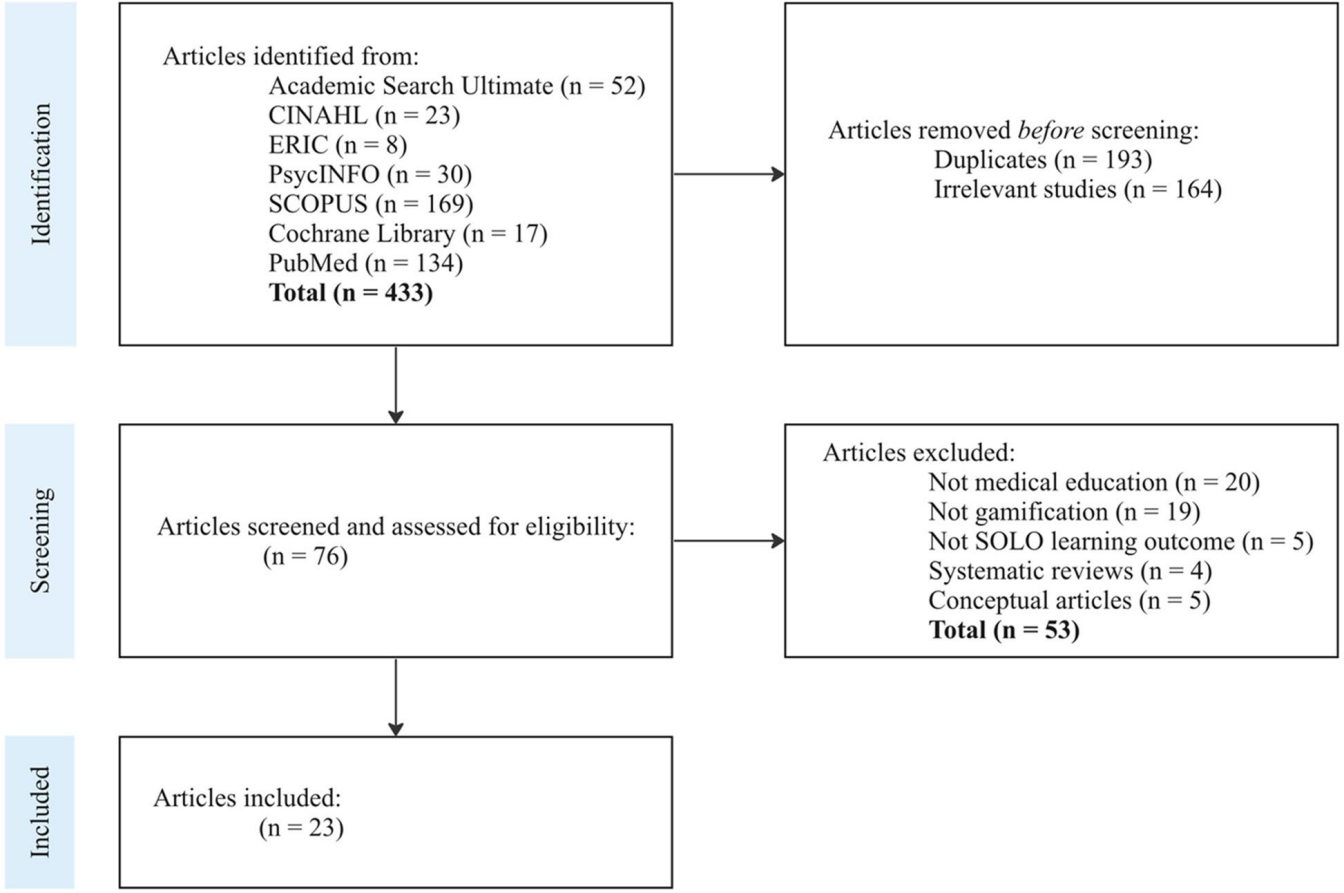
Identification and selection of articles (Adapted from [44] p5)

This study followed the PRISMA systematic literature review process as outlined by Page et al. [44]. See Fig. 1 for the entire search, selection, and review process.

### Search strategy

The research team systematically searched for publications on gamification in medical education. Using multiple databases allowed us to maximize the available data and consider the relevant literature. Consequently, a literature search was conducted using EBSCO (Academic Search Ultimate, CINAHL, ERIC), SCOPUS, PsycINFO, Cochrane Library, and PubMed. The databases were selected for the following reasons: (1) they are highly cited databases in English; (2) Academic Search Ultimate, CINAHL, ERIC, PsycINFO, and SCOPUS are large, multidisciplinary databases, whereas PubMed offers access to more than 35 million citations in the field of life sciences and biomedicine [45], and the Cochrane Library focuses on medicine and other healthcare specialties. This allowed the research team to maximize the available literature and provide a comprehensive overview of the research on medical education and gamification. We used the following two strings of keywords across all seven databases to identify literature of interest: (1) “medical education” AND “innovation” AND “gamification”, and (2) “medical education” AND “gamification.” The literature search was limited to peer-reviewed journal articles published between January 2012 and December 2022.

### Inclusion criteria

The research team included peer-reviewed journal articles published in English on gamification and intended learning outcomes in medical education. Medical education was defined as the training provided during medical school or residency programs. Consequently, literature was included if it specifically focused on the use of gamification in the title, abstract, or keywords, and discussed SOLO learning outcomes in medical education. We only included empirical journal articles, including qualitative and quantitative studies, to meet the objective of systematic reviews by synthesizing research evidence grounded in specific frameworks [46].

### Exclusion criteria

We excluded articles that only describe games or the use of learning technologies in a broader sense to keep the scope of the search process focused on gamification. We also excluded articles that did not discuss learning outcomes following the SOLO taxonomy. Additionally, we excluded research focusing on nursing or pharmacy education as well as all conceptual and systematic review articles. The research team excluded articles published before January 2012 and after December 2022 as well as articles published in languages other than English.

### Study selection

After retrieving the search results from the seven databases, duplicates and irrelevant studies such as conference papers and articles in languages other than English were removed. Abstracts of the remaining articles were screened for preliminary eligibility. In cases of uncertainty, the articles were reviewed in full to determine eligibility according to the inclusion criteria. To strengthen the reliability and validity of the selection process, all the articles were reviewed by at least two members of the research team.

### Data extraction process

The aim of this study is to review the literature on learning outcomes and gamification in medical education in the context of the SOLO taxonomy. Consequently, we extracted data related to gamification and learning outcomes from all reviewed empirical studies. Specifically, the research team first collected data consistent with the keywords relevant to gamified learning and the SOLO taxonomy. Second, the research team reviewed the research questions, results, and discussions in the selected publications.

### Certainty assessment

The research team discussed and addressed any challenges or uncertainties arising during the article selection and data extraction process as a form of triangulation. This further strengthened the validity and reliability of our review findings by ascertaining the consensus of the review results among all research team members.

## Results

The search process yielded a total of 52 publications on EBSCO (Academic Search Ultimate), 23 publications on EBSCO (CINAHL), 8 publications on EBSCO (ERIC), 30 publications on PsycINFO, 169 publications on SCOPUS, 17 publications on Cochrane Library, and 134 publications on PubMed. After removing duplicates and irrelevant studies, 76 publications were selected for screening of preliminary eligibility. Any potential uncertainties in meeting the inclusion criteria were resolved through consensus development among all research team members. Only 23 articles fully met the selection criteria and were included in the study. Figure 1 provides an overview of the study-selection process.

### Application of the SOLO taxonomy

A total of 23 studies described learning outcomes that fall into the SOLO taxonomy. Additionally, three studies were coded with multiple levels of the SOLO taxonomy. In 18 (78%) of the reviewed studies, gamified learning for medical education was designed at a uni-structural level of learning outcomes (see Table 1). In these studies, effective knowledge acquisition within a given time is emphasized in medical education, and gamified methods were applied to motivate learners in this context. For example, [47] developed a gamified web platform to study its impacts on students’ obtaining knowledge about pain neurophysiology and their satisfaction. The gamified methods in this study included providing individual learning paths and quizzes. Two valid questionnaires were used to measure the learners’ knowledge gains. The findings suggest that the gamified platform is effective for both knowledge gain and reducing misconceptions. Multi-structural level learning outcomes were found in three (13%) of the reviewed studies. In addition, three studies (13%) [48–50] identified two or more learning outcomes categorized at the Uni-structural level of the SOLO taxonomy.

**Table 1:** SOLO taxonomy learning outcomes of reviewed studies

SOLO taxonomy level and verbs	Reviewed study (*N* = 23; “*” indicates coded with multiple levels of SOLO)	Research method of the study	Descriptions of SOLO taxonomy learning outcomes in study
Pre-structural: Use irrelevant knowlgde or information for intended learning	None	None	None
Uni-structural: Identify, do, simple procedure (Memorize, identify, recognize, count, define, draw, find, label, match, name, quote, recall, recite, order, tell, write, imitate)	Agudelo et al. [51]	Quantitative	Positive effects on knowledge
	Alexander et al. [52]	Quantitative	Knowledge acquisition/retention
	Aynsley et al. [53]	Qualitative	Feel confident about the subject
	Azhari et al. [54]	Quantitative	Change of leptospirosis knowledge
	Dakroub et al. [55]	Quantitative	Knowledge acquisition
	dos Reis Lívero et al.*[48]	Qualitative	Improve acquisition of knowledge
	Faysal et al. [56]	Quantitative	Knowledge acquisition
	Felszeghy et al. [57]	Quantitative	Knowledge acquisition
	Guérard-Poirier [58]	Quantitative	Performing correct technique/ to identify
	Javed et al. [59]	Quantitative	Knowledge acquisition
	Lee et al. [60]	Quantitative	Factual knowledge
	Nevin et al. [61]	Mixed methods	Knowledge retention
	Scaffidi et al.* [49]	Quantitative	Knowledge/skill retention
	Schlögl et al.* [50]	Quantitative	Describe/Know
	Snyder & Hartig [62]	Quantitative	To recall/memorize
	Tsopra et al. [63]	Mixed Methods	Knowledge acquisition/retention
	Valenzuela-Pascual et al. [47]	Quantitative	Knowledge acquisition
	Van Nuland et al. [64]	Quantitative	Increase academic performance
Multi-structural: Enumerate, describe, list, combine, do, algorithms (Classify, describe, list, report, discuss, illustrate, select, narrate, compute, sequence, outline, separate)	dos Reis Lívero et al.* [48]	Qualitative	Connect between theoretical content
	Schlögl et al. *[50]	Quantitative	Understand (talk about some changes in behavior)
	Vuillaume et al. [65]	Qualitative	Express ideas, knowledge
Relational: Compare/contrast, explain, causes, analyze, relate, apply (integrate, analyze, explain, predict, conclude, summarize, review, argue, transfer, make a plan, characterize, compare, contrast, differentiate, organize, debate, make a case, construct, review and rewrite, examine, translate, paraphrase, solve a problem)	Ali et al. [66]	Quantitative	Understand/practice/ develop and master skills
	Devlin et al. [67]	Qualitative	Comfortability = transfer/make a plan
	dos Reis Lívero et al.* [48]	Qualitative	Develop skills and attitudes
	Pettit et al. [68]	Quantitative	Apply theoretical knowledge to clinical scenarios
	Scaffidi et al.* [49]	Quantitative	Transfer to clinical environment
Extended abstract: Theorize, generalize, hypothesize, reflect (hypothesize, generalize, reflect, generate, create, compose, invent, originate, prove from first principles, make an original case, solve from first principles)	Hudnall & Kopecky [69]	Quantitative	Empathy (reflect, respect/ support, explore)

In five (22%) of the reviewed studies learning outcomes were found at the “Relation” level of the SOLO taxonomy. Gamified learning strategies allow learners not only to attain knowledge, but also to apply knowledge to solve problems. Keywords that illustrate the Relation level in these articles include “understand”, “argue”, “apply”, “master skills”, and “explain”. One study [68] reported that a gamified learning strategy was applied to the technology-enhanced lessons to enhance students’ ability for critical thinking, connecting concepts, and practical application. Applying theories to practice was ranked as the highest learning outcome by students.

Extended-abstract level was found in only one (4%) of the reviewed studies. Hudnall and Kopecky discussed the Extended-abstract level to include empathy as a learning outcome [69]. Specifically, empathy has been considered as a cognitive learning outcome [70]. The study suggested that gamified learning could improve students’ intention to convey empathy, show understanding, and respect emotional expressions.

### Learning outcomes facilitated by motivational and engagement support of gamified learning strategies

It should be noted that six (26%) studies across two SOLO taxonomy levels (Uni-structural and Relational) emphasized improving learners’ engagement with the learning process, their motivation to participate, and their positive self-efficacy through game-based strategies to achieve intended learning outcomes. See Table 2. These factors were measured through learners’ perceptions of gamified learning strategies using self-report questionnaires and post-test scores. The most facilitated learning outcome (four out of six) by supporting learners’ engagement and motivation was the Uni-structural stage. This result suggests an increased need to consider motivational factors in facilitating learning outcome attainment at the Uni-structural level since memorizing, recalling, and defining knowledge might be considered an unappealing learning process by students.

**Table 2:** Studies in which motivation and engagement were considered

Learning outcome keyword	Reviewed Study	SOLO taxonomy
Engagement	Nevin et al. [61]	Uni-structural
Motivation	Valenzuela-Pascual et al. [47]	Uni-structural
Motivation and engagement	Dakroub et al. [55]	Uni-structural
Motivation	Felszeghy et al. [57]	Uni-structural
Self-efficacy	Ali et al. [66]	Relational
Engagement	Pettit et al. [68]	Relational

## Discussion

The reported systematic literature review extends previous inquiries into understanding the effect of gamification on the attainment of learning outcomes in the context of medical education [14, 15, 20]. The findings of this study, to a large extent, augment prior research on medical or health science education on four fronts. First, the present study connects the SOLO taxonomy with gamified learning in medical education. The study finds that the current literature investigating gamified learning in medical education from the SOLO taxonomy perspective lacks empirical inquiries. Since medical education emphasizes the attainment of cognitive learning outcomes in the early years of medical school training, the SOLO taxonomy could provide theoretical and practical instructional guidance to devise comparative or evaluative studies on gamified learning in medical training. Second, within the scope of the SOLO taxonomy, the review suggests opportunities to adopt gamified learning strategies to help medical students attain advanced levels of SOLO learning outcomes according to intended subject matter and levels of medical training. The reviewed studies were primarily focused on the lowest SOLO learning outcome level (18 out of 23, 78%), which could have limited the effects of gamified learning strategies on scaffolding motivating, engaging, immersive, and complex learning processes. Third, similar to prior literature (e.g., [15]), motivational support for gamified learning was identified in the reviewed studies. While motivational support is not the primary learning outcome, it plays a critical role in scaffolding learning outcome attainment at low SOLO taxonomy levels. Finally, combining the SOLO taxonomy’s emphasis on cognitive learning outcomes, gamified learning strategies, and motivational support mentioned in the reviewed studies, our findings offer a preliminary indication in the context of medical education on the empirical relationship between cognitive processing and motivational processing in gamified learning environments [42].

In addition to cognitive learning outcome attainment grounded in SOLO taxonomy and the potential motivational effects of gamified learning, improving soft skills (e.g., communication, team building, collaboration) was identified as an auxiliary learning outcome of gamified learning strategies in reviewed studies. See Table 3. Improving soft skills was found in three out of 23 (13%) reviewed studies across three levels of SOLO taxonomy (Uni-structural, Multi-structural, and Extended abstract). Vuillaume et al. addressed that applying the Escape Room to medical education made students work successfully together and build strong teamwork which is a necessary skill in real medical work environments [65]. In another study [57], the gaming software “Kahoot” was used to teach a histology class to understand its impacts on students’ knowledge gains, learning, and enjoyment. Kahoot consists of multiple-choice quizzes and was tested in five different groups. These groups were different based on the number of times students received Kahoot, whether they received it pre- or post-instruction, and whether students worked on the Kahoot individually or collectively. Findings from this study suggest that not only were the Kahoot gamification features significantly effective for gaining knowledge by recalling the contents, but also pointed out students’ inclination toward team or group gaming over individual gaming. The survey results showed that students who worked in groups received higher scores, and students would like to assess their knowledge gains based on a team approach. The authors indicated that Kahoot was effective in encouraging students’ team-based learning and improving team-based logical skills, which are important for students’ future medical professions.

**Table 3:** Studies in which improving soft skills was supported by gamified learning strategies

Learning outcome keyword	Reviewed Study	SOLO Taxonomy
Collaboration	Felszeghy et al. [57]	Uni-structural
Communication	Hudnall & Kopecky [69]	Extended abstract
Collaboration and communication	Vuillaume et al. [65]	Multi-structural

### Limitations of the study

This present study used multiple highly cited academic publication databases published in English. Nevertheless, the research team recognizes the limitation of not utilizing additional databases as part of the literature sampling frame owing to various definitions associated with gamified learning (e.g., game-based learning, serious games) [20]. The findings of the study are also limited by the research team’s collective interpretations of the mentioned learning outcomes in the reviewed studies, as sometimes such terms were not explicitly presented in the publications. Third, while the SOLO taxonomy is grounded in empirical observations on teaching in various contexts, it lacks well-defined attributes to quantitatively determine which SOLO level(s) the cognitive learning outcome should be. After reviewing the literature and triangulating our analyses, the research team determined the SOLO level(s) of the reviewed studies collectively and qualitatively. Fourth, the research team did not judge the quality or level of the gamified learning during the review process. The alignment with or deviation from the commonly accepted instructional practices of gamified learning in medical education could limit the implications of this study's findings. Finally, the study excluded all publications in 2023 since the publications were ongoing when the study was concluded in late 2023.

## Conclusion

This systematic literature review connects the SOLO taxonomy for cognitive learning outcomes and gamified learning in the context of medical education. The research team reviewed 23 empirical studies qualitatively. The majority of the reviewed studies applied gamified learning for lower levels of SOLO taxonomy learning outcomes, which suggests future research potential to design and implement gamified learning for higher SOLO taxonomy levels for medical education. Motivational support and soft skill development were also considered in the reviewed studies as potential effects of gamified learning on medical education. This emerging trend could inspire future inquiries into exploring gamified learning’s support beyond the scope of cognitive learning outcomes in medical education, including affective and motivational learning.

## Data Availability

All reviewed articles reported in this systematic literature review are listed in the References section for readers to access.
