# Correction: Reflecting on gamified learning in medical education: a systematic literature review grounded in the structure of observed learning outcomes (SOLO) taxonomy 2012—2022

**DOI:** 10.1186/s12909-024-05067-0

**Published:** 2024-02-01

**Authors:** Wenhao David Huang, Viktoria Loidl, Jung Sun Sung

**Affiliations:** 1 Biomedical and Translational Science, Carle-Illinois College of Medicine, Education Policy, Organization, and Leadership, College of Education, University of Illinois Urbana-Champaign, Champaign, IL USA; 2 Education Policy, Organization, and Leadership College of Education, University of Illinois Urbana- Champaign, Champaign, IL USA

**Correction: BMC Medical Education (2024) 24:20**

10.1186/s12909-023-04955-1

Following publication of the original article [1], the authors identified an error in the author name of Viktoria Loidl.

The incorrect name is: Viktoria Loid

The correct name is: Viktoria Loidl
